# Salvage Retzius-Sparing Radical Prostatectomy: A Review of Complications, Functional Outcomes, and Oncologic Outcomes

**DOI:** 10.3390/curroncol29120764

**Published:** 2022-12-08

**Authors:** J. Bradley Mason, Liam Hatch, Christopher Dall, Keith J. Kowalczyk

**Affiliations:** 1 Department of Urology, MedStar Georgetown University Hospital, 3800 Reservoir Rd, Washington, DC 20007, USA; 2 Department of Urology, MedStar Washington Hospital Center, 110 Irving St, Washington, DC 20010, USA; 3 School of Medicine, Georgetown University, 3800 Reservoir Rd, Washington, DC 20007, USA

**Keywords:** prostatic neoplasms, salvage therapy, radiotherapy, urinary incontinence, margins of excision

## Abstract

(Background) Radiation failure for localized prostate cancer is seen in 20–60% of patients who do not undergo extirpative surgery. Though potentially curative, salvage prostatectomy (SS) has not been frequently performed historically due to high rates of complications and postoperative incontinence. With the advent of robotic-assisted radical prostatectomy, these rates appear to be improved. Retzius-sparing approaches have additionally been shown to improve continence outcomes in the index setting, and may further improve continence outcomes in salvage cases while maintaining oncologic integrity. (Methods) We performed a literature review and qualitative analysis of published papers on salvage Retzius-sparing robotic-assisted radical prostatectomy (SRS). Three studies met criteria and were included in analysis. (Results) There were more patients with Gleason Grade Group 1 disease after initial treatment in the SRS group vs. SS (22% vs. 8%). Patients most frequently underwent external beam radiation therapy in both groups (52% vs. 49%). 30-day complication rates were 10% and 26% for SRS and SS, respectively. Continence outcomes were significantly improved in SRS with 59% of continence (based on study criteria) compared to 38% in SS. Time to continence was similarly improved for SRS. Positive surgical margins and biochemical recurrence were not significantly different between SRS and SS in any study. (Conclusions) SRS is a safe and feasible option for salvage treatment of localized prostate cancer and may improve postoperative continence outcomes. Positive surgical margin and biochemical recurrence rates are similar to those reported in SS.

## 1. Introduction

Localized prostate cancer is frequently treated with radiation therapy or brachytherapy, but treatment failure occurs in 20–60% of patients [1,2]. Most patients with biochemical recurrence after RT or BT will begin androgen deprivation therapy which is palliative and carries its own risks including cardiovascular disease and thromboembolism [3], however other salvage treatments are available. A recent meta-analysis reported similarity in recurrence-free survival between all salvage modalities including radical prostatectomy (RP), however a high GU toxicity seen in RP compared to radiation modalities up to 21% [4].

Many cases of BCR after RT or BT are localized to the prostate, and clinical disease takes roughly three to five years to develop in most cases. This leaves an opportunity during which local salvage therapies may offer a curative option [5,6], however historically salvage surgery is performed infrequently due to increased surgical complexity and high complication rates [7,8,9]. These complications include rectal injury, incontinence, and anastomotic stricture, as well as high rates of positive surgical margins and biochemical recurrence. Recent data suggest that lower complication rates may be possible with robotic and even open surgical techniques compared to historical data [7,8,10,11,12,13,14,15,16], making this a more viable option.

The advent of robotic surgery has made salvage radical prostatectomy safer, however incontinence rates remain high [4,7,15,16]. This is thought to be due to sphincter damage during irradiation unmasked by loss of the bladder neck at surgery and may require additional procedures such as artificial urinary sphincter placement [9,12,16]. Retzius-sparing robot assisted radical prostatectomy (RS-RARP) in the index setting has been shown to improve continence outcomes, especially in early return to continence [17,18]. The Retzius-sparing approach in the salvage prostatectomy suggests a theoretical advantage over a true anterior approach in that it offers direct visualization of the rectum during dissection and may allow the surgeon greater ability to avoid rectal injury [19], as well as the preservation of key continence structures. Here, we present a literature review of known studies of salvage Retzius-sparing robotic-assisted radical prostatectomy (SRS) with perioperative, functional, and oncologic outcomes. We hypothesize that patients who undergo SRS will have similar complication rates and oncologic outcomes with improved functional outcomes when compared to standard salvage RARP (SS).

## 2. Materials and Methods

A search of Medline, OVID, and Web of Science with the terms search of “salvage” or “post radiation” and “prostatectomy” and “retzius” and “sparing” or “preserving” yielded six articles. From these six, three had data presented regarding salvage radical prostatectomy with retzius-sparing approach (SRS) and were included in this review. Of those excluded, one was an expert opinion, another was a video abstract only, and two did not study salvage radical prostatectomy. All were retrospective studies comparing SRS to salvage standard radical prostatectomy (SS). Schuetz et al. [20] was a retrospective study of all patients who underwent salvage RARP at a single institution while Madi et al. [19] was a non-randomized prospectively maintained database of cases, studied retrospectively. Kowalczyk et al. [21] was a multicenter, retrospective study of patients who underwent RARP in the salvage setting with either SRS or SS. No comparative analyses were able to be performed in this review given the low number of included studies and potential heterogeneity of subjects across studies according to our statisticians; thus a qualitative analysis was performed. *p*-values shown are reported from individual studies where available.

## 3. Results

### 3.1. Baseline Characteristics

We report qualitative results comparing three case series of SRS and SS in the salvage setting. Table 1 denotes the baseline preoperative characteristics of patients in these studies. In total 81 patients underwent SRS while 45 underwent SS. Median ages and pre-operative PSA values (after index treatment) were similar across groups. Of those reported, there was a higher number of patients with Gleason Grade (GG) 1 disease in the SRS group compared to those who underwent SS (22% vs. 8%). The indices of GG2 (23% vs. 28%), GG3 (18% vs. 28%), GG4 (20% vs. 15%), and GG5 (21% vs. 27%) were similar between groups, and no significant differences were found between initial GG in any studies. These values were taken on biopsy after initial treatment. Schuetz et al. [20] reported initial GG prior to index treatment, thus, these were not included. In terms of initial treatments, patients most frequently underwent external beam radiation therapy (EBRT) in both groups (52% vs. 49%). Brachytherapy was the next most common (21% vs. 27%). Only 1% of SRS patients underwent stereotactic body radiation therapy (SBRT) compared to 7% of SS patients. High-intensity focused ultrasound was more common in SRS (20% vs. 7%). Cryoablation was not common with 2% of SRS and 13% of SS patients choosing this modality. Other treatments were quite rare (4% vs. 2%), with the most common being C-12 ion ablation, used exclusively in the Schuetz et al. [20] series. There were no significant differences between initial treatments in any study.

### 3.2. Perioperative Characteristics

Results are shown in Table 2. Nerve-sparing approaches were done in 27% (22/81) of patients who underwent SRS and 31% (14/45) of patients who underwent SS. Notably, this was not performed in most cases of Schuetz et al. [20] due to oncologic concerns and tissue fibrosis from prior radiation. Most of the patients who underwent nerve-sparing were reported in the Kowalczyk et al. [21] series (19/81 and 14/45). There was only one intraoperative complication reported which was a ureteral injury during SRS diagnosed intraoperatively which required reimplantation. 30-day complications were lower in the SRS group (10%, 6/60) vs. the SS group (26%, 10/39), though this was not significantly different in any study. There was one rectal injury reported in all patients, seen in the SS cohort of Schuetz et al. [20] There was one death within 30 days from cardiac complications in Kowalczyk et al. [21] Mean length of stay was reported in the Schuetz et al. [20] study at 5.7 and 5.4 days and 1 day and 1 day in Madi et al. [19] for SRS and SS, respectively.

Median catheterization time reported in Schuetz et al. [20] was 20.0 days (IQR 13.0–34.0) vs. 27.9 days (IQR 20–96) in SRS vs. SS, respectively (*p* = 0.02). In Madi et al. [19], median catheterization time was reported as 14.0 days (IQR 11.5–17.0) though it is unclear if this is for SRS, SS, or both cohorts. Kowalczyk et al. [21] report a median of 14 days (IQR 9–14) and 33 (IQR 21–45) (*p* = 0.001) for SRS and SS, respectively.

### 3.3. Functional Outcomes

Functional outcomes are seen in Table 3. Continence reporting was variable throughout the three studies. We report continence based on the individual study criteria. Schuetz et al. reported immediate continence after catheter removal (defined as 0 PPD) in 14% (3/12) of RS-RARP patients and 0% (0/7) and 12-month continence rates of 28% (6/14) and 0% (0/7) in SRS and SS patients, respectively. No statistical difference was found between these groups however. They also reported rates of stress incontinence which were also not significantly different between groups.

In Madi et al. [19], continence was defined as 0–1 PPD. 25% (4/16) of patients in the SRS group were continent at time of catheter removal or within 1 month, and 12/15 (80%) achieved continence within three months. No patients in the SS group achieved either metric (*p* < 0.001). Continence at six, nine, and 12 months were similarly improved in SRS though not significantly (*p* = 0.006, 0.004, and 0.384). 100% of the SRS patients achieved continence at the 12-month mark. Time to continence was similarly improved in the SRS group with 90.0 days (IQR 21.7–225.0) vs. 270.0 days (IQR 180.0–454.3) (*p* = 0.0095).

Kowalczyk et al. [21] reported continence as both 0–1 PPD and 0 PPD. They showed significantly improved continence in 0 PPD use for SRS vs. SS (54.1% vs. 6.3%, *p* < 0.001) and improvement in 0–1 PPD use (78.4% vs. 43.8%, *p* = 0.003). Additionally the reported mean PPD (SD) was significantly lower in the SRS group compared to SS (0.57 (0.65) vs. 2.03 (1.81)). Time to continence of 0–1 PPD (days [IQR]) was also improved in the SRS group (47 (30–168) vs. 180 (119–341)).

Potency was only reported by Kowalczyk et al. [21] and found not to be significantly different with 10% (4/40) and 12.5% (4/32) in the SRS and SS groups, respectively. 

### 3.4. Oncologic Outcomes

Table 4 summarizes the oncologic outcomes in these studies. In Schuetz et al. [20], 71% (15/21) of those who underwent SRS had pT2 disease while 29% (6/21) had pT3 disease. In those who underwent SS, 43% (3/7) had pT2 disease while 57% (4/7) had pT3. In Madi et al. [19], patients who underwent SRS had 60% (12/20) pT2 and 40% (8/20) had pT3, while in the SS group 50% (3/6) had T2 and 50% had pT3 (3/6). In Kowalczyk et al. [21], patients with SRS had 50% (20/40) pT2 and 50% (20/40) pT3 disease. In the SS group, 31% (10/32) had pT2 while 69% (29/45) had pT3. None of the differences in final pathology were reported as significant in any study.

In terms of positive surgical margins (PSM), there was heterogeneity in reporting. In Schuetz et al. [20] these were reported as any positive margin as depicted in Table 4. In Madi et al. [19], margin status was split between pT2 (25%, 3/12) and pT3 (37.5%, 3/8) in SRS and (25%, 1/4) and (50%, 1/2). These were further characterized by margin location as bladder neck (50%, 3/6 SRS vs. 0%, 0/2 SS) apical (33%, 2/6 SRS vs. 0%, 0/2 SS), and peripheral (16.7%, 1/6 SRS vs. 100%, 2/2 SS). In Kowalczyk et al. [21], PSM was again separated by pathologic staging. In the SRS group, there were nonfocal PSM in 17.5% (7/40), and focal positive margin in 22.5% (9/40) of pT2, 15% (6/40) of pT3a, and 20% (8/40) of pT3b. In SS, there was nonfocal PSM in 23% (8/32), focal PSM in 18.8% (6/32) of pT2, 31.2% (10/32) of pT3a, and 15.6% (5/32) of pT3b. The rate of PSM in all studies was not significantly different between the two groups.

In terms of BCR after prostatectomy, there were 14% (3/21) vs. 57% (4/7) in Schuetz et al. [20], 20% (4/20) vs. 33% (2/6) in Madi et al. [19], and 23% (9/40) vs. 38% (12/32) in Kowalczyk et al. [21] for SRS and SS, respectively. The rates of BCR were not significantly different in any study. In Schuetz et al. [20], average time to BCR was 12.0 months and 20.3 months for SRS and SS, respectively. In Madi et al. [19], this time was 3.0 months and 3.0 months, respectively. Time to recurrence was not reported in Kowalczyk et al. [21]. Additional treatment after BCR was not reported in Schuetz et al. [20], however one patient in the Madi et al. [19] series underwent additional hormonal treatment and in the Kowalczyk et al. [21] series, five patients in both the SRS (12.8%) and SS (15.6%) groups had adjuvant ADT. This difference, again, was not clinically significant.

## 4. Discussion

Traditionally, salvage RP after radiation therapy has been poorly utilized due to high reported complication rates with open approach as high as 67% [7,22] as well as prohibitive postoperative incontinence as high as 73% [6], urine leak as high as 40% [23], and severe sexual dysfunction. One of the major concerns cited is rectal injury reported as high as 19–28% [7,24] made more likely due to obliteration of tissue planes and fibrosis after radiation [19]. In the years since the advent of RARP, the number of complications in the salvage setting with a standard approach has repeatedly been shown to be improved with reported rates of 39–47% [7,13,25,26], suggesting this a feasible and safe option. In this review of SRS, complication rates seen were much lower than previously reported, and there were zero rectal injuries with this surgical approach. This improvement may be due to the direct visualization of the rectum with the Retzius-sparing approach, which allows for meticulous dissection against the rectal plane. It should also be noted that there was only one intraoperative urine leak in the SRS group in Madi et al. [19] which required conversion to anterior approach to make a watertight anastomosis. This improvement may be due to visualization of the posterior anastomosis with the Retzius-sparing approach. This is a significant improvement in complication rates from prior reports of SS in both open and robotic approaches.

The continence rates for SS of 33–40% [7,15] have been shown to be similar to open salvage RP rates of 45% [7]. In the index setting, RS-RARP has been shown to have improved early return to continence as seen previously reported [17,27]. This is thought to be due to the support of the surrounding ligaments to the anterior urethra which help to maintain sphincteric integrity after SRS [17,27]. Thus, it is possible that these improved continence mechanisms translate to the salvage setting after radiotherapy and other initial treatment modalities. Review of the current available literature suggests significant improvement in immediate and longer-term continence. Additionally, the time to continence remains improved over SS and many patients are immediately continent at catheter removal. Median catheterization time was similarly shorter in the SRS group. However, there were still three patients who underwent SRS in the Kowalczyk et al. [21] series who required postoperative continence procedures, compared to four in the SS group. Two patients in the Madi et al. [19]. series also required artificial urinary sphincter placement postoperatively, however both of these patients underwent SS. Though potency outcomes are poorly recorded throughout the salvage literature they remain consistently poor regardless of the type of surgical intervention. There is likely a potency benefit with nerve-sparing approaches [28], however this is not routinely performed due to concerns for compromising oncologic integrity in the salvage setting. Even with the decreased amount of nerve-sparing approaches reported in these salvage cases, the continence benefits of a Retzius-sparing approach suggest that preserving the supporting structures of the anterior urethra is crucial to continence mechanisms after radiation. There is also evidence that there are a significant number of prostatic nerve fibers on the anterior aspect of the prostate [29], suggesting that leaving these undisturbed in a Retzius-sparing approach may also contribute to improved continence results.

The PSM rates in these three studies are somewhat higher than the 14–31% [7,13,15,28,30] previously reported in other SS series, especially the aggregate rate of 60% for SS. Kowalczyk et al. [21] did specifically report higher rates of PSM which was attributed to a higher percentage of nerve-sparing approaches, though when based on pathologic staging there was no difference in PSM rates between surgical techniques. Schuetz et al. [20] did report significantly lower PSM rates in SRS (*p* = 0.03), though this was compared to conventional open RARP. There were no differences in PSM between robotic approaches. RS-RARP approaches have been previously linked to higher PSM rates [31,32], though it is unclear if these are clinically significant. Indeed, even with this increase in PSM, the rates of BCR and additional treatment were not significantly different from prior reports, suggesting these margins may not have clinical consequences. In Kaffenberger et al. [15], BCR rates of 18% after SS in a retrospective study of 34 patients was associated with shorter PSA doubling time. Eandi et al. [7] similarly reported 33% BCR after SS. However, in all three studies reported here, there was no significant difference between margin locations or relationship to staging.

This review furthers the body of evidence that SRS is a safe and feasible option for curative surgery in patients with recurrent localized prostate cancer. Of course, this study is limited by the paucity of literature surrounding the subject of SRS which did not allow for meta-analysis. There will need to be additional studies comparing these continence results with SS, as well as long term follow up to monitor durable improvement over time. However, with the current evidence, RS-RARP is a safe option in the salvage setting and may improve continence outcomes for these patients.

## 5. Conclusions

SRS is a safe option for treatment of prostate cancer in the salvage setting and can improve continence outcomes in these patients, though potency rates remain low. PSM rates are similar to the standard approach, and BCR rates are not significantly changed. Further study is needed regarding this technique in the salvage setting to ensure oncologic control.

## Figures and Tables

**Table 1:** (a) Preoperative Characteristics. Total number of patients in each study listed as N. Median age (IQR) was 67 years old and 69 years old in RS-RARP and S-RARP, respectively. Median Preoperative weighted PSA was 4.12 ng/mL in RS-RARP and 4.65 ng/mL in S-RARP. Preoperative Gleason grading is shown with total numbers of the study participants with GG1 22%, GG2 23%, GG3 18%, GG4 20%, and GG5 13% in RS-RARP, and 8%, 28%, 23%, 15%, AND 15% in S-RARP. (b) Patterns of local treatment prior to RP presented with individual studies and total N. In RS-RARP, 21% underwent brachytherapy, 52% underwent EBRT, 1% had SBRT, 20% underwent HIFU, 2% had cryoablation, and 4% with other treatment. In S-RARP, 27% underwent Brachy-therapy, 49% had EBRT, 7% had SBRT, 2% with HIFU, 13% with cryoablation, and 2% with other treatment. There were no significant differences in treatment type in any study.

(a): Preoperative Characteristics					
		Study	SRS	SS	*p*–Value
N		Schuetz et al. [20]	21	7	
		Madi et al. [19]	20	6	
		Kowalczyk et al. [21]	40	32	
		Total	81	45	
Age (Median [IQR])		Schuetz et al. [20]	67 (65–71)	69 (61–70)	0.4
		Madi et al. [19]	70 (64–73)	65 (57–68)	0.65
		Kowalczyk et al. [21]	68 (63–70)	66 (60–70)	0.804
		Median weighted	68.2345679	66.3333333	
Median Pre–Op PSA (IQR)		Schuetz et al. [20]	2.5 (1.6–4.4)	5.6 (1.8–6.1)	0.05
		Madi et al. [19]	5.0 (3.2–6.2)	6.5 (3.9–9.4)	0.18
		Kowalczyk et al. [21]	4.6 (2.6–8.3)	4.1 (2.7–6.6)	0.876
		Median weighted	4.12345679	4.65333333	
	**GG1**	Schuetz et al. [20]			
		Madi et al. [19]	4/20 (25%)	1/7 (14%)	
		Kowalczyk et al. [21]	9/40 (23%)	2/32 (6%)	
		Total	13/60 (22%)	3/39 (8%)	
	**GG2**	Schuetz et al. [20]			
		Madi et al. [19]	5/20 (25%)	3/7 (43%)	
		Kowalczyk et al. [21]	9/40 (23%)	8/32 (25%)	
		Total	14/60 (23%)	11/39 (28%)	
Pre–Op Gleason Grade	**GG3**	Schuetz et al. [20]			
		Madi et al. [19]	3/20 (15%)	1/7 (14%)	
		Kowalczyk et al. [21]	8/40 (20%)	8/32 (25%)	
		Total	11/60 (18%)	9/39 (23%)	
	GG4	Schuetz et al. [20]			
		Madi et al. [19]	7/20 (35%)	1/7 (14%)	
		Kowalczyk et al. [21]	5/40 (13%)	5/32 (16%)	
		Total	12/60 (20%)	6/39 (15%)	
	GG5	Schuetz et al. [20]			
		Madi et al. [19]	1/20 (5%)	0/7 (0%)	
		Kowalczyk et al. [21]	7/40 (18%)	6/32 (19%)	
		Total	8/60 (13%)	6/39 (15%)	
	Total	Schuetz et al. [20]			
		Madi et al. [19]			0.71
		Kowalczyk et al. [21]			0.487
**(b): Preoperative Characteristics**					
		**Study**	**SRS**	**SS**	***p*** **–Value**
	**Brachy**	Schuetz et al. [20]	2/21 (10%)	2/7 (29%)	
		Madi et al. [19]	3/20 (15%)	1/6 (18%)	
		Kowalczyk et al. [21]	12/40 (30%)	9/32 (28%)	
		Total	17/81 (21%)	12/45 (27%)	
	**EBRT**	Schuetz et al. [20]	6/21 (29%)	3/7 (43%)	
		Madi et al. [19]	15/20 (75%)	3/6 (50%)	
		Kowalczyk et al. [21]	21/40 (53%)	16/32 (50%)	
		Total	42/81 (52%)	22/45 (49%)	
	**SBRT**	Schuetz et al. [20]			
		Madi et al. [19]	1/20 (5%)	1/6 (18%)	
		Kowalczyk et al. [21]	0/40 (0%)	2/32 (6%)	
		Total	1/81 (1%)	3/45 (7%)	
Local Treatment	**HIFU**	Schuetz et al. [20]	9/21 (43%)	1/7 (14%)	
		Madi et al. [19]	0/20 (0%)	0/6 (0%)	
		Kowalczyk et al. [21]	7/40 (18%)	0/32 (0%)	
		Total	16/81 (20%)	1/45 (2%)	
	**Cryoablation**	Schuetz et al. [20]	1/21 (5%)	0/7 (0%)	
		Madi et al. [19]	1/20 (5%)	1/6 (18%)	
		Kowalczyk et al. [21]	0/40 (0%)	5/32 (16%)	
		Total	2/81 (2%)	6/45 (13%)	
	**Other**	Schuetz et al. [20]	3/21 (14%)	1/7 (14%)	
		Madi et al. [19]	0/20 (0%)	0/6 (0%)	
		Kowalczyk et al. [21]	0/40 (0%)	0/32 (0%)	
		Total	3/81 (4%)	1/45 (2%)	
	**Total**	Schuetz et al. [20]			0.12
		Madi et al. [19]			0.56
		Kowalczyk et al. [21]			0.007

**Table 2:** Perioperative Characteristics. 27% of RS-RARP patients underwent nerve-sparing approaches compared to 31% of S-RARP. Estimated blood loss (EBL) was reduced in RS-RARP. There was 1 intraoperative complication in RS-RARP (ureteral injury) compared to 0 in S-RARP. 30-day complications were 10% in RS-RARP compared to 26% in S-RARP. Median length of stay was similar when reported. Median catheter time was shorter in RS-RARP groups at 20.0 days (IQR 13.0–34.0) vs. 27.9 (IQR 20–96) in Schuetz et al. [20], and 14 days (IQR 9–14) vs. 33 (IQR 21–45) in Kowalczyk et al. [21].

Perioperative Characteristics				
	Study	SRS	SS	*p*–Value
Nerve Sparing	Schuetz et al. [20]	0/21 (0%)	0/7 (0%)	
	Madi et al. [19]	3/20 (15%)	0/6 (0%)	
	Kowalczyk et al. [21]	19/40 (48%)	14/32 (44%)	0.751
	Total	22/81 (27%)	14/45 (31%)	
**EBL (cc)**	Schuetz et al. [20]	300	500	
	Madi et al. [19]	50	100	0.045
	Kowalczyk et al. [21]	100	150	0.039
Intraoperative Complications	Schuetz et al. [20]	0/6 (0%)	0/7 (0%)	
	Madi et al. [19]	0/20 (0%)	0/6 (0%)	1.00
	Kowalczyk et al. [21]	1/40 (3%)	0/32 (0%)	0.368
	Total	1/81 (1%)	0/45 (0%)	
**30–Day Complications**	Schuetz et al. [20]			
	Madi et al. [19]	1/20 (5%)	1/6 (18%)	0.42
	Kowalczyk et al. [21]	5/40 (13%)	9/32 (28%)	0.096
	Total	6/60 (10%)	10/39 (26%)	
**Median Length of Stay (days)**	Schuetz et al. [20]	5.7	5.4	
	Madi et al. [19]	1	1	0.6
	Kowalczyk et al. [21]			
**Median Catheter Time (days) (IQR)**	Schuetz et al. [20]	20.0 (13.0–34.0)	27.9 (20–96)	
	Madi et al. [19]			
	Kowalczyk et al. [21]	14 (9–14)	33 (21–45)	0.001

**Table 3:** Functional Outcomes. Immediate continence after catheter removal was seen in 19% of SRS and 0% of SS. Continence at 12 months was seen in 41% of SRS and 25% of SS, and 59% of SRS at longest follow-up compared to 25% of SS. Potency was 10% in SRS and 12.5% of SS when reported.

Functional Outcomes					
		Study	SRS	SS	*p*–Value
	**Immediate**	Schuetz et al. [20]	3/21 (14%)	0/7 (0%)	
		Madi et al. [19]	4/16 (25%)	0/10 (0%)	
		Kowalczyk et al. [21]			
		Total	7/37 (19%)	0/17 (0%)	
Continence	**12-month**	Schuetz et al. [20]	4/21 (19%)	0/7 (0%)	0.0384
		Madi et al. [19]	8/8 (100%)	4/9 (44%)	
		Kowalczyk et al. [21]			
		Total	12/29 (41%)	4/16 (25%)	
	**At longest follow up**	Schuetz et al. [20]	4/21 (19%)	0/7 (0%)	
		Madi et al. [19]	8/8 (100%)	4/9 (44%)	0.0016
		Kowalczyk et al. [21]	29/40 (73%)	14/32 (44%)	
		Total	41/69 (59%)	18/48 (38%)	
	**Potency**	Schuetz et al. [20]			
		Madi et al. [19]			
		Kowalczyk et al. [21]	4/40 (10%)	4/32 (12.5%)	0.886

**Table 4:** Oncologic Outcomes. 42% of patients had pT2 disease in SRS, compared to 36% in SS. 42% had pT3 disease in SRS compared to 64% in SS. There was a 41% positive margin rate in SRS compared to 60% in SS. Biochemical recurrence (BCR) was seen in 20% of SRS vs. 40% of SS. Time to recurrence was 12.0 months vs. 20.3 months (IQR 3.8–39.0) in Schuetz et al. [20] and 3.0 months (IQR 3.0–5.0) for both SRS and SS in Madi et al. [19] Use of postoperative ADT was 29% vs. 14% in SRS and SS, respectively in Schuetz et al. [20] and 13% vs. 16% in Kowalczyk et al. [21].

Oncologic Outcomes					
		Study	SRS	SS	*p*–Value
	**pT2**	Schuetz et al. [20]	15/21 (71%)	3/7 (43%)	
		Madi et al. [19]	12/20 (60%)	3/6 (50%)	
		Kowalczyk et al. [21]	20/40 (50%)	10/32 (31%)	
		Total	47/81 (58%)	16/45 (36%)	
Path Staging	**pT3**	Schuetz et al. [20]	6/21 (29%)	4/7 (57%)	
		Madi et al. [19]	8/20 (40%)	3/6 (50%)	
		Kowalczyk et al. [21]	20/40 (50%)	22/32 (69%)	
		Total	34/81 (42%)	29/45 (64%)	
	**Total**	Schuetz et al. [20]			
		Madi et al. [19]			0.54
		Kowalczyk et al. [21]			0.027
	**Positive Margins**	Schuetz et al. [20]	4/21 (19%)	4/7 (57%)	
		Madi et al. [19]	6/20 (30%)	2/6 (33%)	
		Kowalczyk et al. [21]	23/40 (58%)	21/32 (65%)	0.482
		Total	33/81 (41%)	27/45 (60%)	
	**BCR**	Schuetz et al. [20]	3/21 (14%)	4/7 (57%)	
		Madi et al. [19]	4/20 (20%)	2/6 (33%)	0.6
		Kowalczyk et al. [21]	9/40 (23%)	12/32 (38%)	0.185
		Total	16/81 (20%)	18/45 (40%)	
	**Time to Recurrence**	Schuetz et al. [20]	12.0 (n/a)	20.3 (3.8–39.0)	
	**Months (IQR)**	Madi et al. [19]	3.0 (3.0–5.0)	3.0 (3.0‚5.0)	
		Kowalczyk et al. [21]			
	**ADT Postoperatively**	Schuetz et al. [20]	6/21 (29%)	1/7 (14%)	
		Madi et al. [19]			
		Kowalczyk et al. [21]	5/40 (13%)	5/32 (16%)	0.735

## Abbreviations

ADT	Androgen-deprivation therapy
BCR	Biochemical recurrence
BT	Brachytherapy
EBRT	External beam radiation therapy
GG	Gleason Grade
PSA	Prostate serum antigen
PPD	Pads per day
PSM	Positive surgical margin
RP	Radical prostatectomy
RT	Radiation therapy
RARP	Robotic-assisted radical prostatectomy
RS-RARP	Retzius-sparing robotic-assisted radical prostatectomy
SBRT	Stereotactic body radiation therapy
S-RARP	Standard robotic-assisted radical prostatectomy
SS	Salvage standard robotic-assisted radical prostatectomy
SRS	Salvage Retzius-sparing robotic-assisted radical prostatectomy
